# Bacteriophages of lactic acid bacteria and their impact on milk fermentations

**DOI:** 10.1186/1475-2859-10-S1-S20

**Published:** 2011-08-30

**Authors:** Josiane E Garneau, Sylvain Moineau

**Affiliations:** 1 Département de biochimie, de microbiologie et de bio-informatique, Faculté des sciences et de génie, Universit&#233 Laval, Quebec city, Québec, G1V 0A6, Canada; 2 Groupe de recherche en écologie buccale, Faculté de médecine dentaire, Universit&#233 Laval, Quebec city, Québec, G1V 0A6, Canada; 3 Félix d'Hérelle Reference Center for Bacterial Viruses, Université Laval, Quebec City, Quebec, G1V 0A6, Canada

## Abstract

Every biotechnology process that relies on the use of bacteria to make a product or to overproduce a molecule may, at some time, struggle with the presence of virulent phages. For example, phages are the primary cause of fermentation failure in the milk transformation industry. This review focuses on the recent scientific advances in the field of lactic acid bacteria phage research. Three specific topics, namely, the sources of contamination, the detection methods and the control procedures will be discussed.

## Introduction

Bacteriophages or, simply, phages (bacteria-infecting viruses) are ubiquitous. They are now acknowledged as the most predominant biological entities on our planet. Up to 10^8^ phages can be found in a single drop of sea water [1]. Scientists have also long tried to use phages (or now phage-derived proteins) to treat diseases such as dysentery or staphylococcal infections [2]. Phages are obligate parasites and most phage multiplication cycle end with cell lysis and the release of hundreds of new virions ready to infect neighbouring cells (for a review on phage biology see [3]). One of the key roles of phages is to balance the bacterial population in every shared environment thereby challenging bacteria to rapidly evolve. Phages can also sometimes turn an industrial microbiologist’s professional life into a nightmare! A biotechnology process that relies on the use of bacteria to produce a molecule or make a product can be disrupted by phages. Problems due to the presence of phages were reported in the food, chemical, pharmaceutical, feed and pesticide industries [4]. However, the dairy industry is probably the one in which phage problems are the most documented.

The manufacture of cheese requires the inoculation of 10^7^ carefully selected bacterial cells (known as starter cultures) per ml of pasteurized milk to control the fermentation and to obtain high-quality end-products. Starter cultures are a combination of various lactic acid bacteria (LAB), usually strains of *Lactococcus lactis*, *Streptococcus thermophilus*, *Leuconostoc* sp., and/or *Lactobacillus* sp. Considering that 10^14^ bacterial cells are needed to produce 1 ton of cheese, it is clear that LAB are of considerable interest to the cheese industry. In the non-sterile environment of raw or heat-treated milk, the added LAB cells will come into contact with virulent phages found in milk [4]. Although phage concentration is usually low in milk, a specific phage population can increase rapidly if phage-sensitive cells are present in the starter culture. The consequent lysis of a large number of sensitive cells will delay or even halt the milk fermentation process leading to low-quality products. In worse cases, the inoculated milk must be discarded. For decades, the dairy industry has been dealing with this natural phenomenon and has relied on an array of control measures, notably adapted factory design, improved sanitation, process changes, specific culture medium, strain rotation, and the use of phage-resistant strains.

The first description of phages affecting a dairy starter culture was reported by Whitehead and Cox in 1935 and since then, the field has seen significant improvements, particularly in the areas of phage genetics, ecology, and resistance to environmental factors [5]. In fact LAB phages are now among the most studied phages. Nevertheless, phage contamination can still occur nowadays leading to product variability and to reduce productivity [6,7]. Phages can also cause problems in the fast growing probiotic field, where the genotype of the strain is highly valuable [8,9].

The topic of “LAB phages” has been *extensively* covered in the past 25 years. Many excellent reviews have tackled LAB phages from various perspectives and readers interested to learn more about this topic will find a list of reviews in additional file 1. Hence, this review will mainly focus on recent scientific advances in the LAB phage research field, with a particular focus on the practical/applied aspects related to the dairy fermentation industry. Topics for which we believe additional research is needed will also be highlighted.

## Sources of contamination

Phages can come from various sources. It is of prime importance to know the potential sources of phages to limit their entry within the manufacturing facilities, which could be deleterious to the fermentation process.

### Raw ingredients

Any raw natural ingredient that enters a fermentation facility may contain phages, albeit at low levels. For example, raw milk, which is an ecological niche for some LAB, is well known to contain phages [6]. Because milk is collected from different farms, phage biodiversity is amplified within milk silos. Since phages can easily propagate in a liquid medium such as milk and since they can also diffuse in gel-like media, only a few sensitive cells are needed to rapidly increase phage levels in a given environment [10]. Using a multiplex PCR method, lactococcal and streptococcal phages have been detected in 37% of the milk samples used for yogurt production in Spain [11], while microbiological approaches demonstrated that 9% of milk samples from various geographical areas in Spain contained *L. lactis* phage [12]. These numbers can be higher in whey samples or final products since phages can propagate during most fermentation processes [12,13]. Titers as high as 10^9^ PFU per ml of cheese whey have been reported [14].

Depending on the frequencies of phage attacks and the size of the facilities, it may be advisable to analyse milk (or other ingredients) for the presence of phages before beginning the fermentation process to confirm that the initial phage load does not represent a significant risk of fermentation failure. If the ingredients are thought to pose a risk, they can be treated to reduce phage levels or used for other processes that will not be affected by phages. Effective cleaning procedures must be also in-place to reduce to the initial phage load.

### Processed or recycled ingredients

The milk fermentation industry may reuse whey proteins to improve the taste or texture of a final product, to increase its nutrient value [15-17], to standardize milk before the fermentation process or to increase the yield [4,18]. Upon whey or milk protein concentration, phages may remain in the whey protein concentrate (liquid or dried) and contaminate the products to which it is added [19]. When using membranes to separate whey components, it is highly possible that phages will be retained by ultrafiltration and/or microfiltration [20]. Depending on whether the retentate or the permeate is used, phages might still be present and cause problems in subsequent transformation processes. Ideally, milk by-products should either be treated to inactivate the phages or be used in a type of fermentation that is driven by different starter cultures. For example, if the whey was collected from a cheddar fermentation made using mesophilic starter cultures, by-products of this whey could safely be used in yogurt manufacture or in a cheese process requiring thermophilic cultures. In addition, the use of concentrated milk products from another dairy plant (which may use different starter cultures) can offer additional protection. Although the latter will also most likely increase the phage biodiversity within the factory.

### Phage reservoir

One perceived source of phages is the starter culture itself. When a temperate phage enters a strain, it can either start the lytic cycle or its genome can integrate into the bacterial chromosome and follow bacterial multiplication. When bacteria carry such a prophage, the cell is called a lysogen. Different bacterial stresses such as heat, salts, antimicrobials, starvation or UV can induce the prophage and trigger the lytic cycle [21,22]. Thus, the use of lysogenic strains in a starter culture may lead to cell lysis during fermentation. Induction can also occur naturally and can reach a frequency of up to 9% [23]. Prophages are carried by many LAB strains [24,25] and often more than one prophage is found in a genome. The most recent analysis revealed that 25 out of 30 commercial, collection or dairy-isolated *Lactobacillus casei*, *Lactobacillus paracasei* and *Lactobacillus rhamnosus* were found to carry inducible prophages [26]. It should be noted that most starter culture suppliers will test their strains for the presence of prophages and their natural induction rate. Usually, lysogenic strains carrying easily inducible prophages will not find their way into commercial products. Of note, phage induction assays cannot be readily performed with undefined starter culture as the exact strain composition of this type of starter is unknown.

The presence of prophage in a strain used as part of a starter culture may not always negatively impact the fermentation process [27]. It has been alleged that prophages have beneficial impact on the organoleptic properties of cheeses through the expression of prophage-encoded endolysins that could stimulate autolysis and the release of intracellular flavor generating-enzymes [28]. Prophage genes also have the advantage of protecting cells against superinfection by other phages, as it was observed in lactococci and *S. thermophilus*[29-32].

Prophages can also act as a reservoir of viral genes and participate in the recombination and release of new virulent phages with enhanced host range and new peculiarities [25,33]. For example, propagation of a virulent phage on a strain carrying a phage resistant mechanism led to the emergence of phage mutants insensitive to the anti-phage system. Comparative analysis of the genomes of the phage mutants revealed that the wild-type phage extensively evolved by large-scale homologous and non-homologous recombinations with the inducible prophage present in the host strain. Thus, natural phage defence mechanisms and prophage elements are contributing to the evolution of the virulent phage population [33]. Another example is the recent isolation of virulent phage infecting a probiotic *L. paracasei* strain which has a similar host range as a mitomycin C-induced phage from another *L. paracasei* strain [26,34].

### Air/surfaces

Whereas phage contamination of milk is probably the most obvious and primary source of phages in a dairy plant, dissemination routes of contaminants can be more complicated to identify. Recently, the presence of airborne lactococcal phages in a cheese plant was investigated because it had been rarely documented [35-37]. A high level of airborne phages in the environment may mean that phage propagation has previously occurred or that phage problems are likely to occur. The large amount of milk processed daily in open cheese vats, as well as whey processing, inevitably lead to liquid splashes and aerosolization of phages [6]. These bacterial viruses can also be aerosolized by air displacement around the surfaces of contaminated fluids and transported elsewhere in the plant. No standard procedures have been established to detect airborne viruses, which can be present in a wide range of particle sizes, from nanometer to micrometer. Hence there is a need for testing sampler and detection methods in a particular environmental setting as each have their advantages and pitfalls (see the review of air sampling in [38]).

Five air samplers were recently tested in a cheese factory and samples were analyzed for the presence of lactococcal phages (936 and c2-like groups) using qPCR. Air sampling was performed for 12 hours next to the filling section at the end of a cheese production line [39]. Results showed that lactococcal phage DNA from both the 936 and the c2 groups could be recovered by every sampler, stressing the importance of these two phage groups in dairy plants. Efficiency in recovering phages as well as their operating mode were variable among the samplers. Still most phage-positive samples, regardless of the sampler, contained at least 10^3^ genomes per m^3^ for both 936 and c2 groups. This shows the omnipresence of phages within fermentation plants and confirms anecdotal evidences that a good ventilation system in a cheese factory is a critical parameter in properly controlling phage dissemination.

Another understudied area is the presence of phages on working surfaces. Not surprisingly, phage genomes of both the 936 and c2 phage groups were also detected at high levels (>10^3^ genome copies/cm^2^) on various equipments and objects found in cheese plants such as door handles, floors, and even on cleaning materials [39]. Although, it is unclear how phages could also find their way into office areas [39], personal might be involved. Even though qPCR cannot distinguish infectious from non-infectious viral particles, this above study demonstrates the importance of good manufacturing and cleaning practices as well as personal training to avoid contamination of the raw or treated products and dissemination of phages.

## Methods of detection

New protocols are still being designed with improved efficiency for detecting phages in industrial processes. Microbiological methods, such as plaque assays or acidification monitoring, have long been the gold standard for phage detection because they are quantitative and sensitive methods (reviewed in [4,40]). Although they are time-consuming, they provide useful data such as the phage’s host range. But new tools can now help to complement microbiological methods so that no compromise is necessary between the specificity, rapidity or phage microbiology data.

### PCR-based methods

Classic PCR detection methods were successfully used to detect or to quickly classify *Lactobacillus*, *Lactococcus* and *Streptococcus* phages [8,11,41-45]. These methods could be used directly on milk or on whey samples to detect the presence of phages. The lowest detection limit reported using a classical one-step PCR method is 10^3^ PFU/ml but this usually varies from 10^4^ to 10^7^ PFU/ml depending on the phages tested and the nature of the sample [8,11,41-45]. Nevertheless, taking into account the time for phage amplification, PCR amplification and gel migration, complete analysis can take several hours. In that regard, qPCR-based methods can overcome this inconvenience by monitoring the replication of specific phage genes in real time, during the fermentation.

qPCR methods were successfully applied to the detection of *Streptococcus thermophilus* phages using different dyes and primers designed to target the gene coding for a minor tail protein, in the case of *pac*-type phages, for the gene coding for the receptor-binding protein for *cos-*type phages [46]. A similar protocol was also developed to detect some *Lactobacillus* phages by targeting a conserved portion of the endolysin gene [47]. qPCR methods provide a fast, specific, and highly sensitive technique to detect phage contamination. However molecular biology techniques can be expensive for routine experiments and can be too specific, meaning that only phages targeted by the primers will be detected. Therefore, PCR-based methods can only be designed if sufficient phage genomic data has been previously made available. Since PCR-based detection methods cannot distinguish infectious from non-infectious phages, microbiological methods can be used in parallel to determine host range and phage titers [48].

### Impedimetric monitoring

Promising new biosensor technologies were recently developed to detect phages. A biosensor to detect whole phages was reported in 2008 [49]. The technique is based on the binding of phages to bacterial cells attached to a chip. A surface plasmon resonance assay detected as low as 10^2^ coliphages/ml of wastewater and their replication could be followed in real time. The same research group later proposed a phage detection assay using impedimetry [50]. The authors demonstrated that it was possible to detect phages using the current variation generated by biofilm degradation in a dairy sample [50]. Still, this latest method had several limitations: i) the host bacteria has to form a biofilm on a chip, ii) only phages specific to the bacteria could be detected, iii) the technique could only assess the presence or absence of the phage and thus was not quantitative. On the other hand, the successful detection of phages using microelectronics opens exciting doors to new types of detection methods.

Garcia-Aljaro and colleagues have also explored a microelectronic technique where a carbon nanotube (CNT)-biosensor was used to detect current changes [51]. The CNT-biosensor is first “functionalized” (by adding 1-pyrene butanoic acid succinimidyl ester), which is then coupled with phage- or bacteria-specific antibodies. The functionalization itself (without any antigens binding) induces an increase in resistance, causing a decrease in current. When antigens, such as phages or bacteria, bind to the antibodies linked to the CNT, the resistance is further increased. The difference between the initial resistance (after functionalization) and the resistance caused by antigens binding is calculated and positively correlated to the incubation time and the concentration of antigens. The more antigens bind to the CNT chip, the greater is the resistance, until it reaches a near-saturation stage. The method was proven to be more effective for phage than bacteria (probably because of their smaller size), meaning that the changes in current could be observed faster. Phages can be detected at a minimum concentration of 10^3^ PFU /ml within just 5 minutes. This promising technology offers key advantages since it is quantitative and the specificity can easily be adapted by the choice of antibody. Since phage proteins are more conserved than DNA sequence, antibodies can be selected to target more than one phage at a time. That said, because of phage diversity, several antibodies will have to be developed to detect the most common phage groups. Still, it is argued that this procedure could be miniaturized and implemented within the routine analysis at low cost [51].

### Flow cytometry

Flow cytometry was used a few years ago to detect the presence of viruses in marine environments using nucleic acid stains. This method can now efficiently enumerates free phages in a sample, regardless of their hosts [52,53]. A novel technique using flow cytometry was also designed to incorporate the host specificity of phages. When phages infect their hosts, these bacteria undergo morphological changes leading to lysis. The loss of mass and the interruption of cell division are two changes that can be monitored by flow cytometry [54]. While low contrast cells can be observed under a phase-contrast microscope following phage lysis, the light scattering of the flow cytometer can efficiently measure the mass of the cells, as long as the bacterial chains (in the case of LAB) are first broken by vigorous shaking. This property allows the flow cytometer to discriminate the infected from the non-infected cells. To assess the presence of phages, the culture is run on the flow cytometer, which gives the distribution of the cells’ mass. A broad distribution of cell mass indicates the presence of both lysed and live cells whereas live cells will typically give a narrow peak. This technique has the advantage of detecting the cell morphology changes regardless of the strain, the phage or the number of strains in a starter culture. The reported detection limit (10^5^ PFU/ml) was comparable to classical PCR methods. It is worth mentioning that flow cytometry was successfully used to detect bacterial morphology changes due to phage infections in a skimmed milk-enriched culture, providing that particles (like eukaryotic cells or fat particles) that could potentially block the cytometer were removed before the assay [54]. From an industrial point of view flow cytometry allows phage detection in real time but requires expensive equipment and trained technicians to perform the assays and analyze the data.

In summary, the choice of the technology will depend on several factors, notably the size of the factory, the quantity of milk transformed each day, the frequency of phage infections, the type of starter culture used, the type of milk fermentation process, the necessity of rapid results, the detection limit, and finally the costs.

## Control

Bearing in mind the ubiquity and the biodiversity of phages within a fermentation facility, it is more attainable to aim for an efficient control of phages rather than a complete elimination. Measures listed below should help reducing the risks of fermentation failure due to phages.

### Sanitation

A good sanitation procedure is certainly one of the key factors in avoiding microbial contaminations and it is also the most efficient way of reducing the spread of phages within the facility. Several sanitizers as well as conditions have been tested on different LAB phages [55-61]. Peracetic acid was often the most efficient biocide while ethanol and isopropanol were usually not effective. Sodium hypochlorite had a variable effect across the studies but was also effective against most LAB phages.

More recently, combinations of biocides were also assayed on *Lactobacillus delbrueckii* phages [55]. Biocides at extreme pH, both high and low, were shown to give the best results, although pH level is not the only factor to take into consideration when choosing a biocide. According to the study, three out of five biocides inactivated 99% of the phage particles in reconstituted commercial non-fat dried skim milk within 2 minutes. Quaternary ammonium chloride, alkaline chloride foam and ethoxylated nonylphenol plus phosphoric acid were the most effective biocides when used at the recommended concentrations (3%; pH 10.5, 2.5%; pH 12.4, and 0.8%; pH 2.0, respectively). On the hand, many of these studies were not performed with commercial products.

New biocides, with different chemical content, are frequently entering the market. While most of them have been tested against bacteria, very few efficiency data are available against phages as well as on the factors (pH, time, temperature, etc.) influencing their activity in milk environment. Ideally, a good sanitizer should be used at most cost effective concentration, have a fast activity (less than 2 minutes for at least 99% inactivation) in the presence of organic materials and have a sanitizing activity against a wide range of LAB phages. Certain biocides can even have residual activity after wiping. Furthermore, because of environmental concerns, the selected sanitizers must be eco-friendly. The materials used in the factory must also be taken into account since some chemicals may interact with the surfaces (e.g. by corrosion). Interestingly European standards have been established for testing virucidal activity of disinfectants used in dairy industry (EN 13610:2002).

Very few data are also available on the efficiency of fumigation/fogging systems, ozone treatment, and UV light irradiation on phages in industrial settings. The mode of action of biocides against phages is also severely understudied.

### Raw material treatment

As indicated above, milk and milk-derived ingredients may contain virulent LAB phages and thus, should be treated to reduce the viral load. Whenever possible, sterile ingredients or media should be used. In the dairy industry, milk pasteurization is the most common practice used to reduce microbial growth and product spoilage.

Heating can greatly reduce the activity of phage particles since it provokes DNA release and changes in phage morphology [62]. It is already documented that many LAB phages will not be inactivated by classical pasteurization procedures [55-61], For example, studies have demonstrated that some 936-like lactococcal phages were resistant to temperatures of up to 97°C for 5 min [14]. Interestingly, it has been suggested that *Lactobacillus helveticus* and *Lactococcus lactis* phages are more resistant to heat treatment than *Streptococcus thermophilu*s and *Lactobacillus delbrueckii* phages [14,61], although this is likely phage-dependent rather than host-dependent. Nonetheless, heat resistance of LAB phages should be monitored closely although the nature of this phenomenon is unknown [14].

A few studies have also assessed the effect of dynamic high pressures on dairy phages [63]. All of them reported a substantial phage reduction using pressures of 100 MPa and higher [63-66]. Müller-Bach and colleagues have also demonstrated that heat and high pressure have a synergistic effect, leading to faster reduction of phage infectivity. Of note, lactococcal 936-like phages were more resistant than c2-like phages [64]. However, phage resistance to heat and dynamic high pressure treatments is highly variable and even differs within a phage group [63,65]. Phages also react differently depending on the medium or the food matrix (milk, whey, milk powder, etc.) [63]. It has often been reported that milk has a protective effect due to the presence of proteins [61,62,64,67], while a higher concentration of salt or fat does not increase the lactococcal phage resistance to heat [19,62,68].

The protective effect of milk proteins on phages reinforces the importance of considering milk or ingredients as potential sources of phages. The physical functionalities of whey proteins can be severely affected by treatments that minimize phage load in the powders [69]. Additional research will help the industry in finding the delicate balance between yield, nutrient content, texture or flavour and phage contamination risks of these ingredients.

### Starter rotation

Starter/strain rotation is probably as old as the use of defined starter cultures and it is still today the cornerstone of an efficient phage control system, especially in a cheese plant, to avoid recurrent amplification of the same phage over consecutive fermentation processes. Still, strain rotation requires a rigorous follow up to detect the emergence of new virulent phages in a cheese plant. Nowadays, the phage species/group of the new phages should also be identified (by PCR) as well as its host range (by microbiological assays) to adjust the strain rotation protocol. In addition, the host range can identify the most phage sensitive LAB strains in a defined starter cultures and perhaps eventually leading to their replacement by unrelated strains. Although this technique is not appropriate for all manufacturing processes, it provides a relatively simple way to minimize fermentation failures due to phages [4].

### Anti-phage mechanisms

To cope with the diversity of phages, bacteria must have varied mechanisms to counteract phage infection. Globally, LAB have developed several systems to overcome infections, each of them targeting a different step in the phage multiplication cycle: i) preventing phage adsorption; ii) blocking entry of phage DNA; iii) cutting phage nucleic acids and iv) aborting the infection (reviewed in [31]). Interestingly, many of these systems are plasmid-encoded and can be moved from one strain to another to increase the general resistance of any given bacterial strain.

In the past few years, a very interesting natural anti-phage system has captured the attention of several research groups. The CRISPR/Cas system, originally found in *Escherichia coli* in 1987, was first shown to provide phage resistance to *S. thermophilus* in 2007 [70-73]. Clustered regularly interspaced short palindromic repeats (CRISPR) loci, along with several Cas (CRISPR-associated) proteins, represent a form of immune system widespread in Bacteria and Archaea. To date, CRISPR loci are found in the genome of many LAB species, namely, *S. thermophilus*, *L. acidophilus*, *L. brevis*, *L. casei*, *L. crispatus*, *L. delbrueckii*, *L. fermentum*, *L. helveticus*, *L. rhamnosus*, and *L. salivarius*. The CRISPR loci evolve through the incorporation of short DNA sequences (spacers), derived mostly from extra-chromosomal DNA such as phage or plasmid sequences, between two partially palindromic repeats. A CRISPR transcript is produced and cleaved within the repeats by Cas protein(s) with or without other host proteins to produce smaller RNAs. These small mature RNAs and Cas proteins guide and cleave in a sequence-specific manner the invading nucleic acids (DNA in the case of *S. thermophilus*) to ensure cell defense [74,75]. This highly efficient system can adapt to gain resistance against virtually any phage. Numerous and detailed reviews on CRISPR/Cas systems have been published lately [76-79].

Abortive infection (Abi) systems, which provide phage resistance through massive cellular death, are also common natural anti-phage mechanisms found in several bacterial genera (reviewed in [31]). Abi systems are very diverse and 23 distinct systems, in *Lactococcus* only, have been described to date [31,80]. One of the most interesting characteristics of Abi systems is that some can function as toxin-antitoxin systems (TA) [81]. As the name implies, TA systems are composed of two tightly controlled regulatory elements: the toxin and the antitoxin which neutralizes the toxin. Perturbation of the balance between these two elements will provoke bacterial cell death. Ongoing fundamental research on the different Abi systems is also increasing our understanding of the precise mechanism behind these variable phage resistance systems and should lead to their optimal utilization. In the past five years, some progress has been made in the understanding of AbiD1 [82], AbiK [83-85], AbiP [86], and AbiZ [87].

The latest discovered lactococcal Abi system is AbiV [88-90]. Contrary to most Abi systems, which are plasmid-encoded, AbiV (201 aa) is chromosomally-encoded. This Abi system is also silent in *L. lactis* MG1363 but can be spontaneously activated due to the reorganization of the promoter region. Interestingly, AbiV can still be naturally transferred from one *L. lactis* strain to another. Whole-genome sequencing of phage mutants insensitive to AbiV revealed a mutation in a gene named *sav* and the polypeptide was named SaV. Overexpression of SaV led to a rapid toxic effect to the cells. Analyses of phage mRNAs and proteins suggested that AbiV blocks the activation of late gene transcription probably by a general inhibition of translation. Using various biochemical approaches AbiV and SaV were found in homodimers and strongly interacting with each other.

Despite all these effort to find and characterize new anti-phage mechanisms, the industrial use of phage resistant bacteria will eventually lead to the emergence of phage mutants able to circumvent the resistance systems. Continuing research on anti-phage systems is still needed to stay one step ahead of phage evolution. Studying phage-host interactions will certainly open new avenues for finding novel anti-phage mechanisms useful in fermentation facilities. For example, it was recently reported that *L. lactis* strain MG1363 is covered by a polysaccharide pellicle that protects the cell against host phagocytosis but also holds a key element for phage-host interactions. Inactivation of the pellicle conferred resistance against a 936-like phage but also led to the formation of abnormally long chains of cocci [91].

The study of spontaneous phage-resistant mutants in different bacterial species has led to the discovery of new phage-interacting proteins. For example, a naturally phage resistant *Roseobacter denitrificans* mutant decreased expression of five membrane proteins and increased expression of several outer membrane proteins [92]. The acquisition of phage resistance in *Staphylococcus aureus* modified the physiological properties of the cell and resulted in decreased expression of virulence genes [93]. In addition, it was recently demonstrated that phage T4 adapted to its host’s growth phase, generating different subpopulations of phages in a lysate. The authors observed that a starved *E. coli* culture had a different phage resistance phenotype than a non-starved culture [94]. A recent genome-scale forward-genetic screen was also performed to find host-dependencies for *E. coli* phage lambda. This screen identified 57 *E. coli* genes, with over half of which have not been previously associated with phage infection, that when knocked out, inhibited the ability of lambda phage to replicate. These results demonstrated a highly integrated phage-host network [95].

### Phage inhibiting components

Different components can be added to create a phage-inhibitory medium with variable efficiency. Purified peptides isolated from a lactococcal phage and added to a culture slightly prolonged the growth of the culture in milk, but did not inactivate the phages [96]. The multiplication of some LAB phages was also shown to be calcium-dependent. The use of phosphates that would sequester divalent cations was hypothesized to reduce the infectivity of phages. However, the results indicated that the use of phosphate in milk at a concentration that did not affect the stability of caseinate particles was not sufficient to reduce the infectivity of all phages tested [97].

Using a GMO approach, the neutralizing capacity of anchored and secreted phage-targeting antibodies was also tested in dairy samples. The gene coding for two different antigen-binding proteins were cloned into *L. paracasei*. Once expressed in the medium, the anchored form of the antibody targeting the phage major capsid protein could inactivate 31% of the phages added to the media. The inactivation rate increased to 86% when using the secreted antibody targeting the receptor binding protein [98].

The use of a combinatorial library of designed ankyrin repeat proteins (DARPins) was also exploited to identify phage specific binding proteins. DARPins are useful as they can be expressed at very high levels and are very stable. Several DARPins that bound specifically to the tip of the receptor-binding protein of a lactococcal phage were selected. Phage infection of *Lactococcus lactis* cells was inhibited by each of the three selected DARPins [99].

Several other GMO approaches have been developed to control LAB phages and they are reviewed in [100].

## Phage classification and biology

Control of phage in industrial facilities also starts with a better understanding of phage ecology. Phage classification schemes are often a matter of debate [101] but they do provide critical information to better control phage infections. Pragmatic classification can usually be universally applied and be helpful to compare phage isolates worldwide. Classification are now available for phages infecting *L. lactis*[102], *S. thermophilus*[103] and *Lactobacillus*[104].

In the past five years, the availability of new DNA sequencing technology at low costs has expedited the characterization of microbial genomes, including LAB phages. The genomes of many reference LAB phages are now available in public databases. It is also anticipated that many additional phage genomic sequences will be made available soon. Comparative genome analysis has confirmed that phage diversification is most often due to the accumulation of point mutations, gene disruption, and recombination [105]. The availability of these phage groupings and genomic sequences should facilitate the characterization of newly isolated phages.

	LAB phage research is now fully engaged into the post-genomic era with the hope of better understanding the phage infection process using integrative strategies. Interest is rising in identifying the complete set of phage genes and proteins involved in the lytic cycle as well as their level of intracellular production using transcriptomic and proteomic approaches. Finally and perhaps one of the most exciting areas is in phage structural biology.

The availability of platforms from structural genomics programs has led to the design of methods to express LAB phage proteins and screen for the best conditions in order to obtain them in a soluble form and prone to crystallization [106]. The recent analysis of the lactococcal p2 phage baseplate structure by X-ray crystallography, electron microscopic analysis and biophysical methods led to a proposed mechanism for the baseplate activation during attachment to the host cell. The baseplate was composed of three protein species, including six trimers of the receptor-binding protein (RBP). When free in solution, the RBPs host-recognition domains [107,108] point upwards, towards the capsid. In the presence of Ca(2+), the RBPs rotated 200 degrees downwards, presenting their binding sites to the host, and a channel opens at the bottom of the baseplate to allow DNA passage. Other comprehensive analysis of various phage proteins have been performed or are underway, with the goal of pinpointing the resemblances within and between functional modules such as connectors, packaging machinery, capsids, tails and baseplates (for a review see [109]). It is expected that in the next few years, other LAB phage proteins or protein complexes will be solved at high resolution, with the perspective to understand mechanisms taking place during phage infection at the molecular level.

## Concluding remarks

Virulent LAB phages are still today a serious industrial concerns and manufacturers are constantly waging war against these viruses to keep them under control. The dairy industry has relied on an array of measures to control this natural phenomenon, including adapted factory design, improved sanitation, process changes, strain rotation, and the use of phage-resistant strains. In spite of these efforts, phages are evolving and new variants keep emerging. Thus, it is essential to find novel control and antiviral strategies to keep up with phage evolution. This research field is now engaged into integrated phage biology approaches to further understand phage diversity and host interactions with the hope of improving the LAB strain selection process and optimizing anti-phage mechanisms.

## List of abbreviations used

Abi: Abortion of infection; DARPins: Designed ankyrin repeat proteins; LAB: Lactic acid bacteria; CNT: Carbon nanotube; CRISPR: Clustered regularly interspaced short palindromic repeats; PFU: Plaque forming unit; RBP: Receptor-binding protein; TA: Toxin-antitoxin.

## Competing interest

The authors declare that they have no competing interests.

## Supplementary Material

Additional file 1 **List of reviews on phages and their relation to LAB.** The reviews addressing mostly phages in relation to LAB, and written from 1980 to 2011 were listed here. Reviews were classified in 3 categories but do not belong exclusively to one category. Only reviews written in English were listed. Click here for file
