# Supplementary material for: Bacteriophages of lactic acid bacteria and their impact on milk fermentations
Source: Microb Cell Fact. 2011 Aug 30;10(Suppl 1):S20. doi: 10.1186/1475-2859-10-S1-S20 (PMC3231927; doi:10.1186/1475-2859-10-S1-S20)
Supplement: Additional file 1 — List of reviews on phages and their relation to LAB. The reviews addressing mostly phages in relation to LAB, and written from 1980 to 2011 were listed here. Reviews were classified in 3 categories but do not belong exclusively to one category. Only reviews written in English were listed. [file 1475-2859-10-S1-S20-S1.doc]

| **Additional file 1.** **List of reviews on phages and their relation to LAB** | | | | | |
| --- | --- | --- | --- | --- | --- |
| **Category** | **Year** | **Type** | **First author** | **Title** | **Reference** |
| **Phages of LAB** | 1983 | Review | Teuber, M. | The bacteriophages of lactic acid bacteria with emphasis on genetic aspects of group N lactic streptococci |  |
|  | 1984 | Book chapter | Klaenhammer, T. R. | Interactions of bacteriophages with lactic streptococci |  |
|  | 1988 | Review | Séchaud, L. | Bacteriophages of lactobacilli |  |
|  | 1989 | Review | Jarvis, A. W. | Bacteriophage of lactic acid bacteria |  |
|  | 1990 | Review | Davidson, B. E. | Temperate bacteriophages and lysogeny in lactic acid bacteria |  |
|  | 1991 | Review | Jarvis, A. W. | Species and type phages of lactococcal bacteriophages |  |
|  | 1995 | Mini-review | Sable, S. | The lysins of bacteriophages infecting lactic acid bacteria |  |
|  | 1996 | Review | Gasson, M. J. | Lytic systems in lactic acid bacteria and their bacteriophages |  |
|  | 1996 | Review | Schouler, C. | Genomic organization of lactic acid bacteriophages |  |
|  | 1998 | Review | Brüssow, H. | Molecular ecology and evolution of *Streptococcus thermophilus* bacteriophages |  |
|  | 1999 | Book chapter | Brüssow, H. | Phages of *Streptococcus thermophilus* |  |
|  | 2001 | Review | Brüssow, H. | Phages of dairy bacteria |  |
|  | 2001 | Review | Boucher, I. | Phages of *Lactococcus lactis*: an ecological and economical equilibrium |  |
|  | 2002 | Review | Desiere, F. | Comparative genomics of phages and prophages in lactic acid bacteria |  |
|  | 2002 | Review/News | Moineau, S. | Phages of lactic acid bacteria: from genomics to industrial applications |  |
|  | 2002 | Review | Mullan, M. | Bacteriophages for lactic acid bacteria [On-line] |  |
|  | 2007 | Book chapter | Vegge, C. S. | Bacteriophage-host interaction in lactic acid bacteria |  |
|  | 2009 | Review | Villion, M. | Bacteriophages of *Lactobacillus* |  |
|  | 2010 | Book chapter | Rodríguez González, A. | Bacteriophages of lactic acid bacteria |  |
|  | 2010 | Review | Quiberoni, A. | *Streptococcus thermophilus* bacteriophages |  |
| **Category** | **Year** | **Type** | **First author** | **Title** | **Reference** |
| **Phages in relation to the industry** | 1980 | Review | Ogata, S. | Bacteriophage contamination in industrial process |  |
|  | 1987 | Book chapter | Sanders, M. E. | Bacteriophage of industrial importance |  |
|  | 1989 | Review | Wunsche, L. | Importance of bacteriophages in fermentation processes |  |
|  | 1994 | Book chapter | Sanders, M. E. | Bacteriophages in industrial fermentation |  |
|  | 1996 | Book chapter | Neve, H. | Bacteriophages |  |
|  | 2000 | Review | Marks, T. | Bacteriophages and biotechnology |  |
|  | 2001 | Review | Coffey, A. | Traditional and molecular approaches to improving bacteriophage resistance of Cheddar and Mozzarella cheese starters |  |
|  | 2002 | Review | Heap, H. A. | Bacteriophage in the dairy industry |  |
|  | 2004 | Review | Los, M. | Bacteriophage contamination: is there a simple method to reduce its deleterious effects in laboratory cultures and biotechnological factories? |  |
|  | 2005 | Book chapter | Moineau, S. | The control of bacteriophages in food fermentation |  |
|  | 2006 | Book chapter | Bogosian, G. | Control of phage in commercial microbiology and fermentation facilities |  |
|  | 2007 | Book chapter | Émond, É. | Bacteriophages and food fermentation |  |
|  | 2008 | Review | Callanan, M. J. | Bacteriophages in industry |  |
|  | 2009 | Review | Ahn, S. I. | The characteristics, detection and control of bacteriophage in fermented dairy products |  |
|  | 2010 | Book chapter | Labrie, S. J. | Bacteriophages in industrial food processing: incidence and control in industrial fermentation |  |

| **Category** | **Year** | **Type** | **First author** | **Title** | **Reference** |
| --- | --- | --- | --- | --- | --- |
| **Phage defence systems** | 1987 | Review | Klaenhammer, T. R. | Plasmid-directed mechanisms for bacteriophage defense in lactic streptococci |  |
|  | 1988 | Review | Sanders, M. E. | Phage resistance in lactic acid bacteria |  |
|  |  |  |  |  |  |
|  | 1993 | Review | Hill, C. | Bacteriophage and bacteriophage resistance in lactic acid bacteria |  |
|  | 1994 | Book chapter | Klaenhammer, T. R. | Bacteriophages and bacteriophage resistance |  |
|  | 1995 | Review | Dinsmore, P. K. | Bacteriophage resistance in *Lactococcus* |  |
| **Phage defence systems** | 1996 | Review | Daly, C. | Biotechnology of lactic acid bacteria with special reference to bacteriophage resistance. |  |
|  | 1998 | Review | Allison, G. E. | Phage resistance in lactic acid bacteria |  |
|  | 1999 | Review | Moineau, S. | Applications of phage resistance in lactic acid bacteria |  |
|  | 1999 | Review | Forde, A. | Bacteriophage defence systems in lactic acid bacteria |  |
|  | 2002 | Review | Coffey, A. | Bacteriophage-resistance systems in dairy starter strains |  |
|  | 2002 | Review | McGrath, S. | Bacteriophage-derived genetic tools for use in lactic acid bacteria |  |
|  | 2004 | Review | Sturino, J. M. | Bacteriophage defense systems and strategies for lactic acid bacteria |  |
|  | 2005 | Review | Chopin, M.-C. | Phage abortive infection in lactococci: variations on a theme |  |
|  | 2006 | Review | Sturino, J. M. | Engineered bacteriophage-defence systems in bioprocessing |  |
|  | 2010 | Review | Labrie, S. J. | Bacteriophage resistance mechanisms |  |

The reviews addressing mostly phages in relation to LAB, and written from 1980 to 2011 were listed here. Reviews were classified in 3 categories but do not belong exclusively to one category. Only reviews written in English were listed.

# References
